# Stepped-Tube Backside Cavity Piezoelectric Ultrasound Transducer Based on Sc_0.2_AI_0.8_N Thin Films

**DOI:** 10.3390/mi15010072

**Published:** 2023-12-29

**Authors:** Xiaobao Li, Haochen Lyu, Ahmad Safari, Songsong Zhang

**Affiliations:** 1 School of Microelectronics, Shanghai University, Shanghai 200444, China; 2 Shanghai Melon Technology Company Ltd., Shanghai 201899, China; 3 Department of Materials Science and Engineering, Rutgers, The State University of New Jersey, Piscataway, NJ 08854, USA

**Keywords:** PMUT, directivity, acoustic field, scandium-doped aluminum nitride (Sc_0.2_AI_0.8_N)

## Abstract

This paper presents a novel piezoelectric micromachined ultrasonic transducer (PMUT) with theoretical simulation, fabrication, and testing. Conventional methods using a PCB or an external horn to adjust the PMUT acoustic field angle are limited by the need for transducer size. To address this limitation, the stepped-tube (expanded tube) backside cavity PMUT has been proposed. The stepped-tube PMUT and the tube PMUT devices have the same membrane structure, and the acoustic impedance matching of the PMUT is optimized by modifying the boundary conditions of the back cavity structure. The acoustic comparison experiments show that the average output sound pressure of the stepped-tube backside cavity PMUT has increased by 17%, the half-power-beam-width (θ_-3db_) has been reduced from 55° to 30° with a reduction of 45%, and the side lobe level signal is reduced from 147 mV to 66 mV. In addition, this work is fabricated on an eight-inch wafer. The process is compatible with standard complementary metal oxide semiconductor (CMOS), conditions are stable, and the cost is controllable, plus it facilitates the batch process. These conclusions suggest that the stepped-tube backside cavity PMUT will bring new, effective, and reliable solutions to ranging applications.

## 1. Introduction

Piezoelectric Micromachined Ultrasonic Transducers (PMUTs) have gained significant attention in recent years. Conventional bulk piezoelectric transducers are gradually being replaced by Micromachined Ultrasonic Transducers (MUTs) due to their large size, incompatibility with CMOS processes, the requirement for acoustic matching layers, and other limitations [1]. Based on the sensing mechanism, MUTs can be categorized into two types: capacitive ultrasonic transducers and piezoelectric ultrasonic transducers. These transducers are thin-film in nature and have their acoustic impedance matched to the medium (acoustic or aqueous), eliminating the need for a matching layer. Compared to CMUT, PMUT does not require a bias current to operate, resulting in significantly improved device reliability. In the last decade, researchers have started conducting comprehensive research on PMUT. PMUT has been widely used in fingerprint identification, gesture recognition, medical imaging, flow meters, distance measurement, humidity sensors, and other applications [2,3,4,5,6,7,8,9]. 

Currently, various piezoelectric materials such as lead zirconate titanate (PZT), barium calcium zirconate titanate ceramics (BCZT), and aluminum nitride film (AIN) have been widely applied [8,10,11]. However, the preparation of PZT requires a high temperature environment (>480 °C) due to the lead content in the process, which is gradually being replaced in today’s environmentally conscious context. BCZT is a lead-free piezoelectric material, but its preparation also requires higher temperatures [11]. Compared to PZT and BCZT, the lead-free piezoelectric material AIN can be deposited at lower temperatures (<300 °C), offering improved receiving sensitivity and temperature stability. It is characterized by a wide range of raw material sources and compatibility with CMOS processes [12]. Compared to pure AIN, the piezoelectric performance of ScAIN significantly improves while retaining many of the characteristics of AIN, such as being easy to deposit, etch, and being lead-free [13]. Therefore, in this paper, Sc_0.2_Al_0.8_N film-based PMUT is chosen for preparation.

In the process of ultrasonic propagation, there is attenuation and dissipation of sound wave energy. In order to enhance the detection distance and accuracy, a highly directional radiation mode is required. In addition to the main lobe, traditional PMUT also has side lobes during operation. The presence of side lobes can cause detection errors and other problems, such as incorrectly identifying other objects or echoes from the ground. Therefore, increasing the output sound pressure and improving the directivity of the sound field have been the focus of research for some time. Nayak et al. [14] received the radiation pattern of a single PMUT coupled with air by adjusting the PCB package size. Haolin Yang et al. [15], with an external horn, optimized the sound field angle of a 66 kHz PMUT array from 100° to 20° with a half-power-beam-width (θ_-3db_). Yuchao Zhang et al. [16] optimized the acoustic output of a single PMUT at a resonant frequency of 91 kHz with an external speaker. Scott et al. [17] at the University of California, Davis, optimized the device structure, MEMS process wafer, and CMOS process wafer level bonding to achieve an optimized back pressure recovery structure. This resulted in a 4.5 dB increase in sound pressure level and improved directionality. However, the side lobe level also increased accordingly. External PCB packaging and speakers will increase the size of the device, thereby negating the advantages of PMUT’s small integration. By connecting an external speaker and adjusting the PCB package size, although the directivity of the PMUT improves and the sound pressure level increases, it does not significantly suppress the generation of side lobes.

In this paper, a new structure PMUT based on Sc_0.2_AI_0.8_N is theoretically analyzed, designed, simulated, prepared, and characterized. The height of the back cavity is controlled by stepwise etching. A stepped-tube backside cavity (expanded tube backside cavity) is introduced at the back of the PMUT. This cavity serves to inhibit the generation of side lobes while also enhancing the directivity of the acoustic field. The purpose of this is to focus the output energy more effectively, increase the acoustic pressure output, and regulate the angle of the sound field for the PMUT. 

## 2. Design and Modeling

### 2.1. Theoretical Analysis

**Figure 1:**
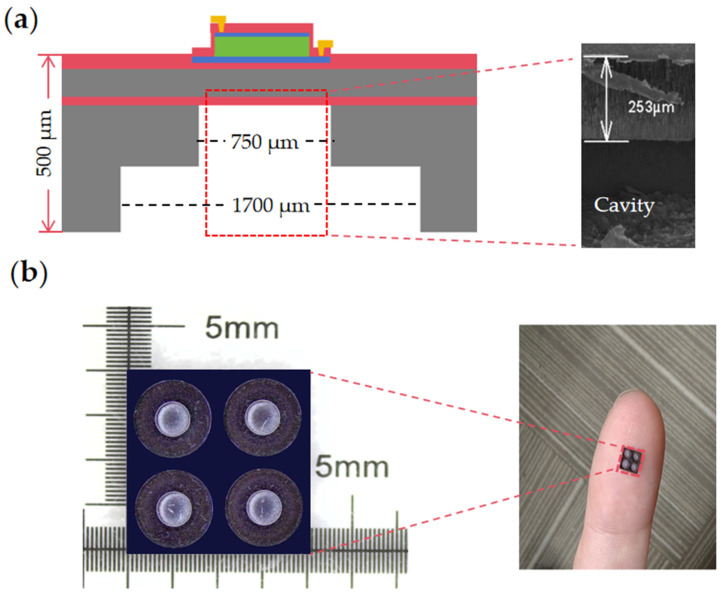
(**a**) Proposed PMUT, 2D structure schematic, and SEM backside cavity height localized view. (**b**) Optical microscopic top view of the backside cavity structure.

The structure of the new stepped-tube PMUT, as reported in this paper, is shown in Figure 1, and the membrane structure consists of an Mo/Sc_0.2_Al_0.8_N/Mo stack with thicknesses of 0.1 μm/1 μm/0.2 μm on an SOI wafer, respectively. The difference lies in the structure of the backside cavity. The overall height of the two backside cavities in the PMUT is 500 μm. The conventional structure is a tube backside cavity with a diameter of 750 μm. The stepped-tube backside cavity is formed by two Deep Reactive Ion Etching (DRIE) to create an expanding tube resonance cavity at the bottom of the tube resonance cavity, with a height of 250 μm and a diameter increment of 950 μm.

According to an analysis of acoustic theory, the radiation impedance describes the process of matching the impedance of sound waves as they propagate from the diaphragm into the surrounding medium. When a sound source vibrates, it generates a sound field in the surrounding medium, and at the same time the source is also immersed in the radiated sound field in which it is situated. Therefore, it will also be subject to a certain reaction, which is equivalent to a force impedance being applied to the original mechanical vibration system. The real part is equivalent to mechanical damping, reflecting the existence of energy loss, vibration, and a sound source that will radiate sound energy [18]. The virtual component of the performance of the inertial force, which is equivalent to the sound source itself on the mass of the additional radiation mass and vibration operates to overcome the inertial force and perform work, thereby storing energy in the system [19].

**Figure 2:**
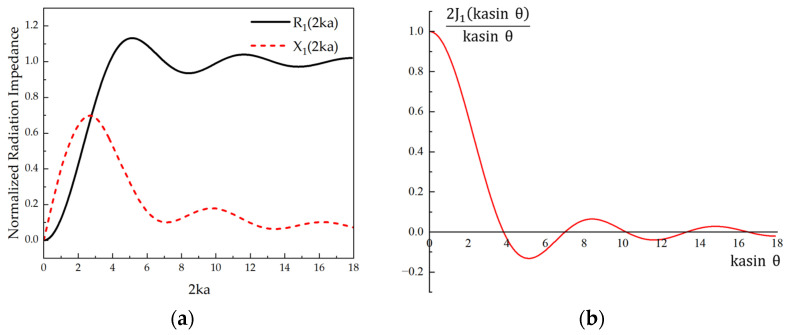
(**a**) Radiative impedance real part R_1_ (2ka) and imaginary part X_1_ (2ka) normalized to the characteristic impedance in air. (**b**) Normalized plot of the directivity function D (a, θ).

The horn consists of tubes with varying cross-sectional areas. Its advantage is that it can provide almost any acoustic impedance. The expression for the acoustic impedance of the horn throat is (1) [18]:

(1) $$Z_{a0}=\frac{\rho_{0}c_{0}}{S_{0}}\left[\frac{Z_{al}cos\left(\gamma l+\theta \right)+j\frac{\rho_{0}c_{0}}{S_{l}}sin\gamma l}{\frac{\rho_{0}}{S_{l}}cos\left(\gamma l-\theta \right)+jZ_{a}sin\gamma l}\right]$$

where S_0_ is the opening area at the end of the horn, ρ_0_ is the density of the medium, c_0_ is the propagation speed of sound in the medium, l is the length of the horn, Z_al_ is the acoustic impedance at the exit of the horn when ka is larger, the horn can be used as an approximation for the radiated acoustic impedance of the piston on the infinite baffle, and the change of the opening area of the horn can increase the radiated power while making the directionality of the sound source clearer and the energy more centralized [20]. The acoustic impedance of the PMUT, Z_pmut_, and the radiated impedance, R_r_, is calculated as in (2) and (3) [3].

(2) $$Z_{pmut}=\frac{\rho_{0}c_{0}}{A_{m}}\left(1-\frac{{2J}_{1}\left(2ka\right)}{2ka}+j\frac{{2K}_{1}\left(2ka\right)}{2ka}\right)$$

(3) $$R_{r}={\left(\pi a_{eff}^{2}\right)}^{2}R_{1}$$

where J_1_ and K_1_ are the first-order Bessel and first-order Struve functions, and the radiation impedance is normalized as shown in Figure 2a [21], where the real part R_1_ is the radiation resistance, which determines the energy coupled to the medium by the PMUT, the imaginary part X_1_ is the radiation resistance, ρ_0_ is the density of the medium, c_0_ is the propagation speed of the acoustic wave in the medium, k is the number of waves, a and a_eff_ are the radius and the effective radius of the PMUT. A_m_ is determined by the radius of the opening at the end of the backside cavity. The average radiated power $\bar{W_{r}}$ can be obtained as (4) [20].

(4) $$\bar{W_{r}}=\frac{1}{2}R_{r}u_{a}^{2}$$

(5) $$f=\frac{1.63}{r^{2}}\sqrt{\frac{D}{\sum \rho_{i}t_{i}}}$$

It can be observed that when the vibration speed of the sound source remains constant, the average radiated power value is influenced by the radiation impedance and the equivalent radiated area. The equivalent radiated area is determined by the number of waves and the effective radius of the device, which in turn depends on the radius of the opening at the end of the backside cavity. To increase the radiated power, it can be achieved by either increasing the effective radius of the device or increasing the frequency of the PMUT. From Equation (5), it can be seen that the frequency on the backside is usually inversely proportional to the square of the diaphragm radius [22]. The stepped tube on the backside cavity increases the equivalent radiant area of the PMUT while maintaining the frequency almost unchanged.

According to the Rayleigh principle, the sound pressure of PMUT at any point P(r, θ) in the far field as in (6) [18]:

(6) $$P(r,\theta )=j\omega \frac{\rho_{0}u_{a}a^{2}}{2r}\left[\frac{2J_{1}\left(kasin\theta \right)}{kasin\theta }\right]e^{j\left(\omega t-kr\right)}$$

D(a, θ) is a spatial distribution function that describes the radiated free far sound field. It characterizes the distribution of the acoustic field emitted by the transducer, the degree of concentration of the radiated power, the accuracy of the localization, and the discriminatory ability. Combined with P(r, θ), the normalized directivity function of the circular thin-film transducer is expressed as (7) [23]:

(7) $$D(a,\theta )=\left|\frac{2J_{1}\left(kasin\theta \right)}{kasin\theta }\right|$$

As shown in Figure 2b, related to the directivity function are the wave number k and the equivalent radius a_eff_ of the PMUT. Since the environment in which the device operates is deterministic, the speed of sound in the medium c_0_ does not change. Therefore, what affects the directivity of a single-array transducer are the PMUT operating frequency and the equivalent radius a_eff_.

From D(a, θ), it can be seen that as the operating frequency of the PMUT increases, the 2ka increases and the directivity improves [18]. However, the corresponding side lobe also increases, and the sound pressure attenuation in the medium is inversely proportional to the transmission distance X as the frequency rises. Therefore, increasing the frequency is not a good choice for improving the directivity of a single array element. Similarly, when the radius of the PMUT is larger, the directivity improves. However, the number of side lobes also increases gradually. Therefore, we aim to enhance the directivity of the single-array element by appropriately increasing its equivalent radius.

### 2.2. Modeling Analysis

**Figure 3:**
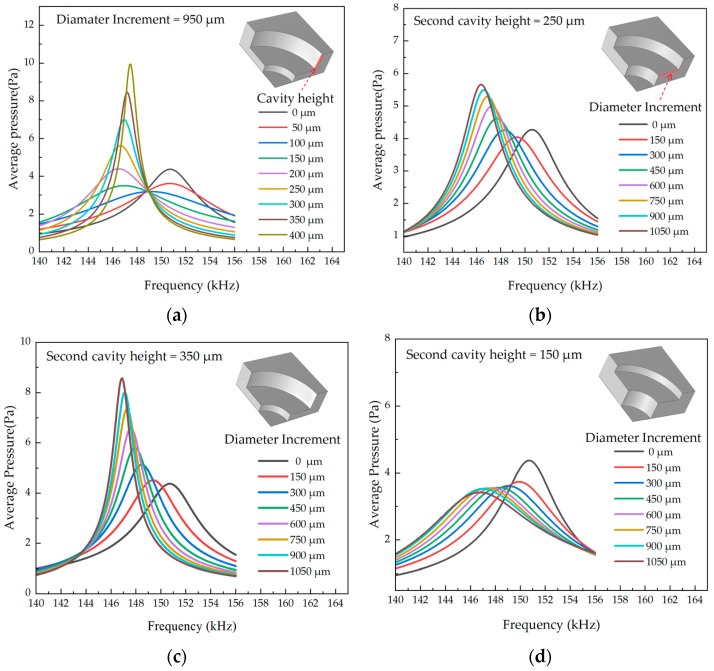
Average sound pressure results of FEM simulation at 3λ below the PMUT resonance cavity: (**a**) D_i_ = 950 μm, varying the height of the dilatation tube; (**b**) H_c_ = 250 μm, varying the incremental diameter of the dilatation tube; (**c**) H_c_ = 350 μm, varying the incremental diameter of the dilatation tube; (**d**) H_c_ = 150 μm, varying the incremental diameter of the dilatation tube.

In order to determine the structure of the backside cavity of the PMUT, the dynamic response of the PMUT is modeled using FEM (COMSOL 5.6) simulation software. The piezoelectric layer is made of Sc_0.2_AI_0.8_N film doped with 20% Sc and has a thickness of 1 μm. The main body of the elastic layer is made of top silicon and has a thickness of 4 μm. The height of the backside cavity is 500 μm. The average value of the acoustic pressure is measured at a location 3λ underneath the backside cavity. The results are shown in Figure 3d [24]. When the height of the second layer backside cavity (H_c_) is small, the PMUT is considered to have a tube sound pressure gain by (500 − H_c_) for the backside cavity height. Increasing the diameter of the second layer backside cavity does not result in sound pressure gain. This is because the reflected sound waves from the backside cavity wall and the PMUT output acoustic wave are reduced when they are superimposed, leading to a decrease in the output sound pressure [23]. From Figure 3a–c, when Hc is more significant, the backside cavity sound pressure gain directly benefits from the diameter increment of the second layer. This is because the effective area increases, resulting in an increase in radiated power when the diameter increment D_i_ = 950, the cavity height H_c_ = 400, and compared with the pipe PMUT, the average sound pressure at 3λ below the stepped pipe backside cavity is improved by 120%. For a single PMUT, the larger backside cavity end opening obtains a larger effective radiating area, which can produce higher bulk velocity and higher sound pressure output, push more air, and convert other forms of energy into the acoustic energy of air vibration.

**Figure 4:**
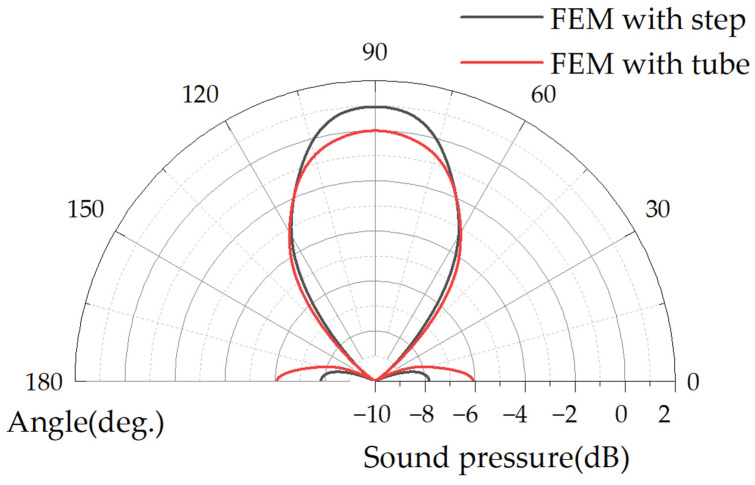
FEM simulated far-field SPL directivity normalized plot: red line is tube PMUT with 0 diameter increment at the end of the cavity; black line is stepped-tube PMUT with 950 μm diameter increment at the end of the cavity and 250 μm second backside cavity height.

Considering the process conditions and area limitations, the work in this paper produces PMUT cavity diameter increment D_i_ = 950 μm and expansion tube cavity height H_c_ = 250 μm, as well as FEM simulation geometry and far-field output average sound pressure, as shown in Figure 3a. The expansion tube PMUT below the 3λ measured an average sound pressure gain of 27%, Q_tube-3db_ = 34, Q_step-3db_ = 47. The FEM far-field SPL directivity normalization results are shown in Figure 4. The main lobe SPL sound field angle constriction and the side lobe level decreases, the stepped-tube PMUT side lobe compared to the primary lobe intensity decreased by 9 dB, while the tube PMUT side lobe is only reduced by 6 dB compared to the main lobe. The fabrication of the device for this paper will be presented in the third part.

## 3. Fabrication

**Figure 5:**
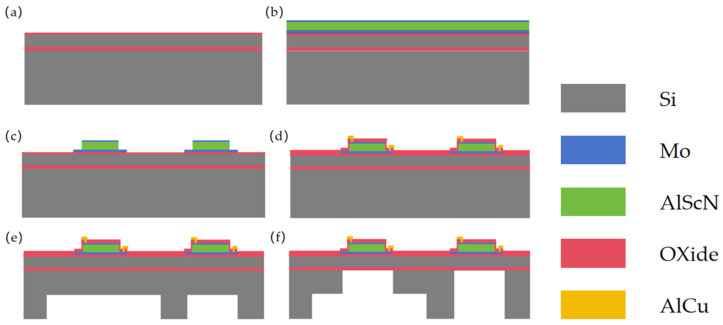
Process flow for fabrication of ultrasonic transducers with stepped-tube backside cavities based on Sc_0.2_AI_0.8_N films on eight-inch wafers: (**a**) starting from SOI wafers; (**b**) deposition of Sc_0.096_AI_0.904_N/Mo/Sc_0.2_AI_0.8_N/Mo stacks; (**c**) top Mo, Sc_0.2_AI_0.8_N, bottom Mo, dry etch patterning of the seed layer, Sc_0.096_AI_0.904_N; (**d**) PECVD deposition, through-hole via etching, aluminum-copper alloy deposition and etching for PMUT metallization interconnections; (**e**) backside large cavity 250 μm DRIE silicon etching; (**f**) backside tiny cavity silicon and SiO_2_ etching to release the membrane.

**Figure 6:**
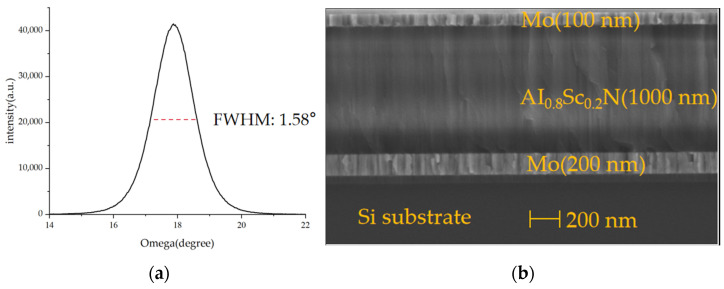
(**a**) XRD rocking curve measurement of Sc_0.2_AI_0.8_N (002) peak; (**b**) SEM scanning electron microscopy of Mo/Sc_0.2_AI_0.8_N/Mo film stack.

As shown in the process flow in Figure 5, an eight-inch silicon-on-insulator (SOI) wafer, with a top silicon thickness of 4 μm and a buried oxygen layer of 500 nm, is used for this work. First, a 300 nm layer of SiO_2_ is deposited on the surface with PECVD as a dielectric layer. Before the first deposition, the wafer needs to undergo pretreatment at a high temperature of 450 °C for 5 min under vacuum. This process is designed to remove impurities and water vapor adhered to the SOI wafer [25]. Then, a one-step deposition of Sc_0.096_AI_0.904_N/Mo/Sc_0.2_AI_0.8_N/Mo stacked layers was carried out. The XRD and SEM results are shown in Figure 6. The full width at half-maximum (FWHM) value of 1.58° indicates that our deposited Sc_0.2_AI_0.8_N films have good crystalline quality, in which Sc_0.096_AI_0.904_N serves as the seed layer, which is favorable for the subsequent deposition of highly oriented films with the (002) orientation. This reduces the surface roughness of the Sc_0.2_AI_0.8_N film [16]. The resulting piezoelectric coefficients for the Sc_0.2_Al_0.8_N samples were d_33_~9 pm/V and d_31_~−4 pm/V by the cantilever method. The seed layer, Mo electrode layer, and Sc_0.2_AI_0.8_N were all deposited using the Sigma^®^ Standard PVD (Sigma^®^ Deposition System from SPTS, Newport, UK). Pure Mo targets were used for the deposition under the Ar atmosphere, and an AI_0.8_Sc_0.2_ alloy target was used for the deposition of the Sc_0.2_AI_0.8_N layer under the Ar/N_2_ atmosphere.

To optimize the etching slope angle and serve as a hard mask, a 400 nm layer of SiO_2_ was deposited by PECVD before Sc_0.2_AI_0.8_N etching, and the main etching process was carried out under a boron BCl_3_/Cl_2_/Ar atmosphere. In order to optimize the etching rate and improve etching homogeneity, we utilized the Inductively Coupled Plasma etching (ICP, Omega^®^ etch system from SPTS, Newport, UK). Before the main etching process, we employed boron BCl_3_/Ar gas for pretreatment. The native ScAIN oxide can be effectively removed by a short exposure to BCl_3_/Ar plasma [26]. This is difficult to remove with the main etching gas on the film’s surface, and it also increases the etching rate. It helps to reduce the problems of product yield caused by over-etching [27]. Then, a 200 nm SiO_2_ layer is deposited, and vias are etched to create interconnections between the bottom and top electrodes.

After metallized interconnects are etched, the wafer is flipped to the backside. Finally, the film is released using Deep Reactive Ion Etching (Rapier RP, Omega^®^ etch system from SPTS, Newport, UK). In order to achieve a high depth-to-width ratio in deep silicon etching, the process dimensions of the PMUT stepped resonance cavity are achieved using the Bosch process to etch the bottom diameter of the stepped-tube backside cavity, which has a diameter of 1700 μm [28]. The substrate height is controlled at 250 μm through step-by-step etching. This is followed by PECVD deposition of a 400 nm SiO_2_ as a hard mask on the backside of the wafer. The purpose of this deposition is to prevent the photoresist from melting due to the increase in wafer temperature caused by warpage. Warpage causes a gap between the wafer and the etch chamber platen, which can interfere with the second small resonance cavity etching process. The goal is to maintain optimal etching conditions. Finally, in order to improve the adhesion of the photoresist to the etched backside cavity, the backside was initially treated with HDMS (hexamethyl-disilazane), then coated with photoresist (C9005), and a secondary photolithography process was performed on the backside cavity with a diameter of 750 μm using a double-sided aligned lithography machine (SUSS, MA/BA8 Gen3). Then, the 250 μm SI was etched, and finally a 500 nm buried oxygen layer was etched to release the membrane.

## 4. Experiments

### 4.1. Electrical Characterization

**Figure 7:**
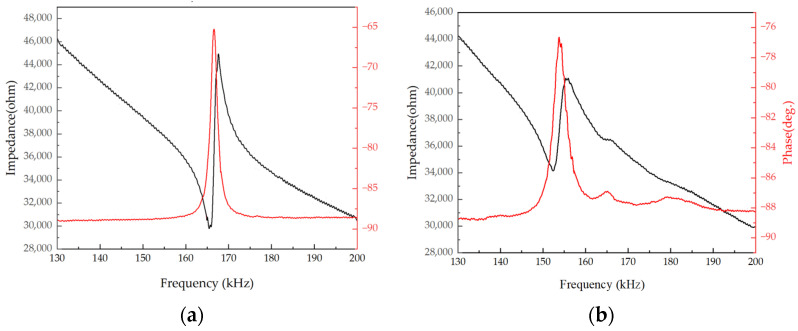
Device characteristics: (**a**) Tube PMUT electrical impedance characteristics; (**b**) Stepped-tube PMUT electrical impedance characteristics.

An impedance analyzer measured the electrical parameters of the PMUT, and Figure 7 shows the electrical characteristics of a single PMUT, a tube backside cavity, and a stepped-tube backside cavity device, respectively.

The discrepancy between the frequencies obtained from electrical tests and simulations can be attributed to the frequency shift caused by the stresses introduced during the deposition of the Mo/Sc_0.2_AI_0.8_N/Mo stacked films. The inclusion of the stepped resonance cavity alters the boundary conditions of the film in the vertical direction, resulting in a decrease in the stiffness of the structural layer. This leads to a reduction in the frequency of the PMUT in the stepped-tube backside cavity.

### 4.2. Acoustic Characteristics

**Figure 8:**
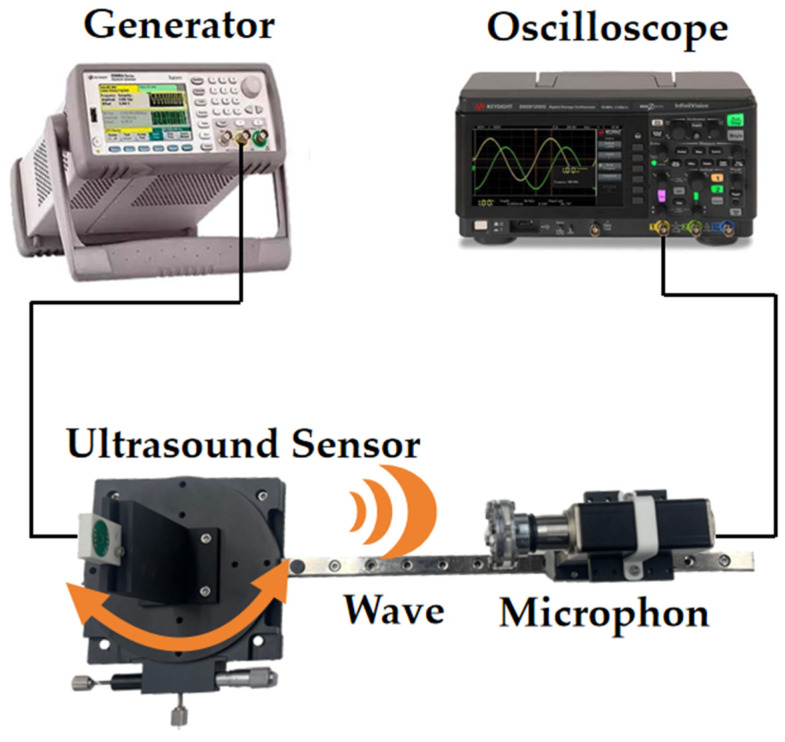
Schematic diagram of the angle experiment environment.

**Figure 9:**
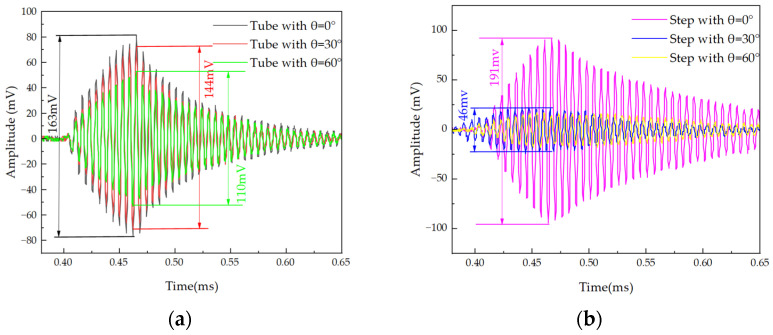
Device characterization for performance at different angles: (**a**) tube backside cavity PMUT transmitting characteristics); (**b**) stepped-tube backside cavity PMUT transmitting characteristics.

The transmission performance of two PMUT structures was compared using the experimental setup shown in Figure 8. The experimental setup consisted of a rotating platform for PMUT, a function generator, a digital storage oscilloscope, a standard commercial microphone (CM16/CMPA, 2–200 kHz, Avisoft Bioacoustics Co., Ltd., Glienicke/Nordbahn, Germany), and a scale to measure shift distance. The PMUT was fixed onto the platform base, and the distance and angle between the sensor and the microphone could be adjusted by moving and rotating the bottom platform. During the experiment, the microphone was positioned 20 cm in front of the PMUT. The PMUT was driven by a function generator at its resonant frequency. The function generator produced 10 Vpp pulsed continuous square waves with a period of 10 cycles. The ultrasonic waves emitted by the PMUT propagated through the air and were received by the microphone. The received waves were then displayed on an oscilloscope. By rotating the circular bottom platform, the center axis of the PMUT and the microphone is set at 0°, 30°, and 60° when the acoustic time-domain characterization is shown in Figure 9. In the same experimental environment, the acoustic energy at the front end of the PMUT in the stepped-tube backside cavity was more concentrated. The acoustic pressure was increased by 17% compared to that of the tube backside cavity. Compared to when the microphone and the PMUT were at 0°, the amplitude of the measured signal in the stepped-tube backside cavity were reduced by 75% and 81% when the PMUT was positioned at angles of 30° and 60°, respectively. In contrast, the amplitude signal of the tube backside cavity structure is only reduced by 11% and 32%.

After receiving the excitation signal, the PMUT vibrates and emits ultrasonic waves accordingly. This vibration does not stop immediately, but rather it gradually becomes calm after the initial vibration with the help of the elastic layer. The period between the end of the initial vibration and the steady state is referred to as the ringing time [4]. The ringing time affects the range of the blind zone. Taking the transceiver as an example, the time difference between the input signal and the reflected wave is used to measure the distance of the detected object. When the ringing time is too long, it results in an overlap of the reflected signal and the ringing time, which affects the signal resolution [29]. The acoustic time-domain signals from the stepped-tube backside cavity and the tube backside cavity PMUT are transformed by the FFT to obtain the BW_tube-3db_ of 5.16 kHz and the BW_step-3db_ of 3.34 kHz. The acoustic quality factor Q is calculated as 31 and 49, which is consistent with the simulation results. The structure of the backside cavity adjusts the impedance matching of the device. The stepped-tube backside cavity PMUT radiates more energy into the air at the same time. Therefore, acoustic measurements result in a higher quality factor Q compared to impedance measurements. But it will also bring adverse effects. For example, when it is used to measure distance, the echo signal from close objects will sometimes be obscured by the trailing signal.

**Figure 10:**
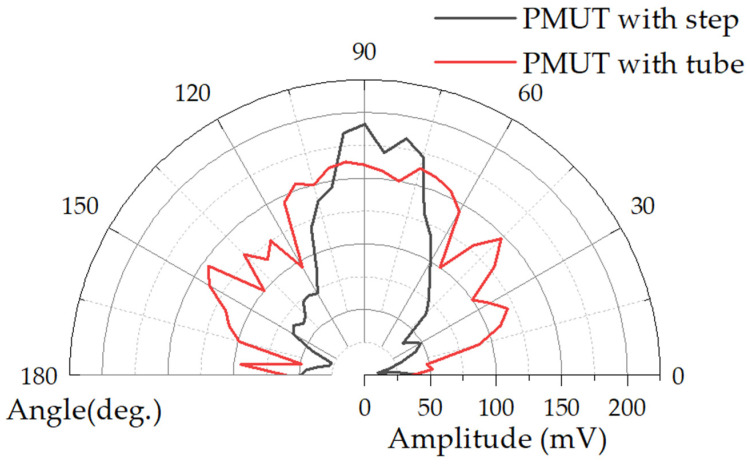
Measured far-field sound field angle of PMUT with tube and stepped-tube backside cavities.

Measurements were continued using the experimental conditions shown in Figure 8. These commercial microphones are relatively accurate across a range of frequency responses and do not require recalibration. The ultrasonic signal was measured every 5° rotation of the bottom platform in the same experimental environment. The results of the directivity of the device are shown in Figure 10. The half-power-beam-width (θ_-3db_) of the tube backside cavity is approximately 55°, with two high secondary peak side lobes. These lobes reduce the sound pressure signal by 10% and 26%, respectively, compared to the main lobe. As a result, there is dispersion of acoustic energy and a decrease in the sound pressure of the PMUT in a single direction. The black curve represents the actual measurement of the stepped-tube resonant cavity, showing a half-power-beam-width (θ_-3db_) of 30°. The introduction of the stepped-tube backside cavity reduces the half-power-beam-width (θ_-3db_) of the PMUT by 45%. This reduction is achieved without significant generation of high secondary side lobes. Additionally, the sound pressure signal is reduced by 65% and 77%, respectively. Compared to that of the main lobe, the side lobe amplitude signal is reduced from 147 mV to 66 mV. When the PMUT is excited, the lack of isolation between the transducers leads to electrical, acoustic, and structural coupling. This coupling generates parasitic and spurious modes in the surrounding PMUT, which can interfere with the normal operation of the oscillator elements and even the entire PMUT array. This interference is known as crosstalk [30,31]. The stepped-tube PMUT has a smaller side lobe level, which theoretically reduces the array crosstalk effect caused by acoustic coupling in this study. The smaller acoustic field angle can help prevent echo interference in the non-detection direction during the ranging process.

## 5. Conclusions

In this paper, we demonstrate the performance of a stepped-tube PMUT based on a Sc_0.2_Al_0.8_N piezoelectric film. According to the FEM simulation results, reducing the side lobe level of the PMUT can be achieved by increasing the cavity height (H_c_) and the diameter increment (D_i_) of the second backside cavity layer. Taking advantage of the large back-cavity structure eliminates the need for a complex PCB package or a large external horn. The effective radiating area is increased, resulting in improved acoustic output sound pressure. Acoustic experiments verified this conclusion, showing a 17% increase in average sound pressure at a distance of 20 cm, while reducing the half-power-beam-width (θ_-3db_) from 55° to 30°. The level of side lobe reduction for the emitted signal was 81%, while the reduction in the emitted signal from the tube resonant cavity PMUT was only 32%. The stepped-tube PMUT achieves a highly directional sound field and a lower side lobe level. The above experiments show that the stepped-tube backside cavity structure offers a new optimization approach for ScAIN MEMS PMUT devices, specifically for range proximity sensing and reducing array crosstalk effects.

## Data Availability

The data are available within the article.
